# Portable continuous wave Doppler ultrasound for primary healthcare in South Africa: can the EUnetHTA Core Model guide evaluation before technology adoption?

**DOI:** 10.1186/s12962-021-00261-z

**Published:** 2021-02-15

**Authors:** Debjani Mueller, Robert C. Pattinson, Tsakane M. Hlongwane, Reinhard Busse, Dimitra Panteli

**Affiliations:** 1 Department of Health Care Management, Berlin Institute of Technology, Berlin, Germany; 2 Charlotte Maxeke Research Cluster, Johannesburg, South Africa; 3 Department of Obstetrics and Gynaecology, School of Medicine, Faculty of Health Sciences, University of Pretoria, Pretoria, South Africa; 4 Research Centre for Maternal, Fetal, Newborn and Child Health Care Strategies, University of Pretoria, Pretoria, South Africa; 5 Maternal and Infant Health Care Strategies Research Unit, South African Medical Research Council, Pretoria, South Africa

**Keywords:** Health technology assessment, Doppler ultrasound, South africa

## Abstract

**Background:**

This study had a threefold aim: to test the value of stakeholder involvement in HTA to reduce evidence gaps and interpret findings; and to assess a medical device by applying the EUnetHTA Core Model (CM) in South Africa and thus ultimately provide a first overview of evidence for potential widespread adoption of the technology in a primary health care (PHC) setting. Used in primary healthcare setting for obstetric use, the technology under assessment is a low-cost continuous wave Doppler ultrasound (DUS).

**Methods:**

The scoping of the assessment was defined by involving policy makers in selecting the domains and corresponding questions relevant to the ultrasound and its use. Additionally, hospital managers were invited to respond to dichotomous questions on the criteria for procurement. To substantiate evidence obtained from an initial literature review, different stakeholders were identified and consulted. The evidence generated fromall steps was used to populate the high-ranked assessment elements of the CM.

**Results:**

The HTA on continuous-wave DUS incorporated the evidence on organizational, ethical, and social value of its use together with effectiveness, safety, and cost-effectiveness of the technology. The domains on “health problem” and “safety” had a higher rank than the rest of the nine domains. Unexplained fetal mortality is the largest single contributor to perinatal deaths in South Africa. Pregnant women in PHC setting were examined using a continuous-wave DUS, after their routine antenatal visit. The healthcare professionals interviewed, indicated the benefit in the use of continuous-wave DUS in the PHC setting and the need for training.

**Conclusions:**

Collection and generation of evidence based on the HTA CM and the chosen decision criteria provided a generalized but structured guidance on the methodology. Several questions were not applicable for the technology and the context of its use and elimination of those that are inappropriate for the African context, resulted in a pragmatic solution. Engaging and consulting local stakeholders was imperative to understand the context, reduce evidence gaps, and address the uncertainties in the evidence, ultimately paving the way for technology adoption. Given the ongoing studies and the evolving evidence base, the potential of this technology should be reassessed.

## Background

Globally, maternal and child mortality remain unacceptably high, despite significant progress in recent years [1]; reducing them remains a priority for many governments. Particularly in sub-Saharan Africa (SSA), access to adequate essential reproductive health services remains one of the challenges towards achieving universal health coverage (UHC). Maternal mortality amounted to 533 deaths per 100,000 live births in 2017, compared to 11 deaths per 100,000 live births among high-income countries and 211 deaths per 100,000 live births globally [2]. The third-trimester stillbirth rate in SSA is approximately 10 times higher than in developed countries—29 vs 3 per 1000 births [3]. The *Every Newborn Action Plan* to end preventable deaths, endorsed by the United Nations member states at the World Health Assembly in 2014, set a stillbirth target of 12 per 1000 births or less by 2030. SSA countries are still far from achieving this goal [3].

In many resource-poor settings, the availability and quality of care in health facilities are not sufficient to hinder adverse maternal or fetal outcomes [4, 5]. While the use of conventional ultrasound diagnostics is a routine component of antenatal care in high-income countries, in many low- and middle-income countries (LMICs), the only way to determine fetal growth rate at the primary care level is by palpation and measuring the symphysis fundal height (SFH) using tape measure [6]. Small studies in LMICs have shown that the use of the conventional ultrasound directly influences antenatal care (ANC) utilization, improving referral for detected conditions and gestational age dating, and increasing the use of hospital for deliveries [7]. However, Goldenberg et al. [7] in their cluster randomized trial, concluded that routine conventional ultrasound found no effect on ANC attendance, reduction in maternal, stillbirth or neonatal mortality. Furthermore, it seems to contribute to the improvement of patient management and the confirmation of clinically suspected obstetric complications [8]. The potential impact of routine ultrasound use in LMICs where perinatal and maternal mortality rates are high, and access to and quality of antenatal care are poor [7, 9] can be significant. The Doppler ultrasound (DUS), a specific type of ultrasound detects changes in the pattern of fetal blood flow in the umbilical artery [10]. Continuous-wave Doppler, a type of DUS is relatively inexpensive, easier to use and has lower energy output than pulsed-wave DUS. However, continuous wave Doppler does not provide precise depth information. This can be mitigated by using pulsed Doppler instruments, which has the capability of depth resolution and measuring a variable sample volume. It is however, more costly and requires trained sonographers. A Cochrane systematic review of the application of the DUS in high-risk pregnancies suggests that its use can decrease the risk of perinatal death resulting in fewer obstetric interventions [11].

A system-based approach focusing on improving the quality of care in health facilities and ensuring the delivery of essential healthcare services is necessary to reduce maternal and perinatal mortality in LMICs [12]. In South Africa, primary healthcare (PHC) is offered free of charge to the population by the state [13], while all health services are free of charge for pregnant women and children under the age of six. The district health system, divided into 52 health districts, is the government’s mechanism to facilitate and strengthen the delivery of primary care and district hospital services [14]. District and sub-district Management Teams and hospital Chief Executive Officers (CEOs) are responsible for services at communities and facilities in their districts [15]. In this capacity, they have to ensure that safe, effective, and essential technologies are procured and maintained within their respective budgets. Indeed, while the public health sector accounts for about 40% of all expenditure on health, it is under pressure to deliver services to about 80% of the population [16]. Inequalities exist in the distribution of hospitals between provinces and rural settings, with healthcare workers being disproportionately employed in the private sector [13]. It was therefore essential to consider options for improving ANC that would reflect the limited overall resources in the public health system (financial and professional) and the need for flexibility to address the unequal distribution of services in the country.

With this in mind, the South African Medical Research Council (SAMRC), Tygerberg Research Unit in Cape Town, and Centre for Scientific and Industrial Research (CSIR) developed a novel portable continuous-wave DUS, the Umbiflow™, to be used in remote areas and PHC settings. Currently, the technology is undergoing CE tests for market access, and in collaboration with World Health Organization (WHO), is tested in various cohort studies in South Africa and abroad.

Health Technology Assessment (HTA) is used to evaluate the potential for adoption of the Umbiflow™ technology. HTA enables evidence-based decision-making on the introduction and use of safe, effective, and efficient technologies at various levels of the healthcare system. Its methodology has been developed substantially over the past twenty years; for instance, the European Network for Health Technology Assessment, (EUnetHTA) Core Model™ provides a comprehensive framework for conducting HTA. The Core Model (CM) was developed in the context of co-operation between European countries. It provides a validated and preference-oriented structured methodology for the assessment of health technologies [17]. South Africa, similar to other settings that have recently adopted HTA, does not yet have a methodological guideline to evaluate non-pharmacological technologies. However, the importance of HTA for improving quality and safety of PHC services was also noted at a Presidential Summit on UHC [18] in 2018.

In emerging settings, and especially when the technology under investigation is not yet fully established, it is necessary to consider a broad range of evidence to obtain a full picture of the consequences of the technology’s implementation. To increase the acceptance of the HTA findings, involving the relevant stakeholders throughout the HTA process is beneficial, beginning with the scoping of the assessment [19, 20].

This study aims to:

- Evaluate a technology, the Umbiflow, which is at an earlier stage of adoption, by application of the EUnetHTA Core Model in a non-European setting;
- Demonstrate the value of stakeholder engagement and consultation to mitigate evidence gaps, address uncertainties, and interpret findings when evaluating technologies in an emerging setting with limited resources;
- Provide a first overview of evidence for the potential widespread adoption of Umbiflow™ in PHC in South Africa.

## Methods

**Fig. 1:**
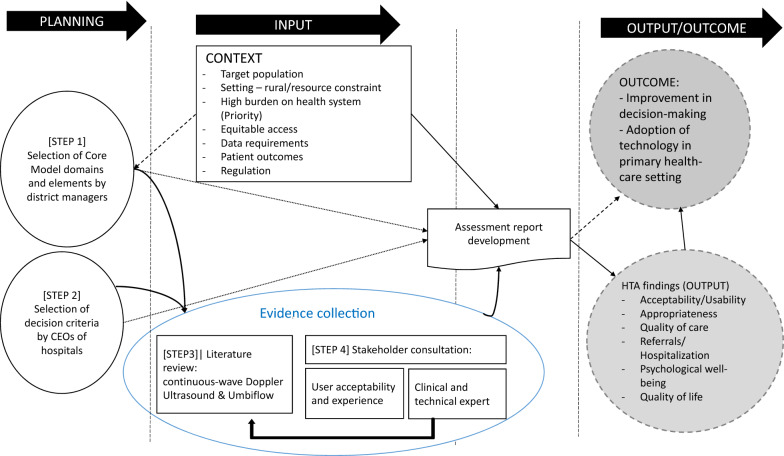
The data collection and extraction for HTA of the Umbiflow™

The CM, which enables integration of various dimensions of a technology (Fig. 1) and the impact of its use, was chosen to guide a stakeholder-based assessment of Umbiflow™. To define the scope of the HTA, district managers responsible for the delivery of primary care services, were invited to rank the relevance of the CM domains and the corresponding questions (Step1) relevant to the technology. A modified survey was developed to establish consensus on the decision criteria used by hospital managers to procure technologies (Step 2). In Step 3, evidence on continuous-wave DUS was collected from the literature and consolidated with the initial search and recommendations arising from Step 4. The initial search accompanied the scoping of the assessment and for formulating the research question. Step 4 involved consultation with clinical and technical experts and collating information on user experience from questionnaires. Each step is described below in more detail.

### Step1: Selecting Core Model domains and issues for consideration

Considering that, the application of HTA for medical devices (MDs) at the PHC level in South Africa is nascent, applying a validated standardized HTA methodology for MDs can provide a framework for collecting and synthesizing information and sharing of results [21]. The CM contains issues, topics and domains, which are nested together into assessment elements (AEs). It is a preference-oriented model composed of nine evaluation domains (Fig. 1). Each domain represents “a wide framework within which the assessed technology is considered. It provides an angle of viewing the use, consequence, or implications of using the technology and any other aspects applicable to it” [22]. The domains provide guidance on tools and methods for the systematic identification, analysis, and presentation of information. Each domain is divided into several topics and each topic is further divided into various issues; the latter correspond to specific questions to be examined when assessing a technology [22]. Because of the novelty of using HTA in general, and the CM in particular, CM for evaluating MDs in South Africa and the limitations on data, a prioritization of domains and AEs was undertaken by the decision-makers to identify their perceived evidentiary needs.

District health managers were invited for a workshop on June 6, 2019, at the University of Pretoria to define the scope of HTA for this case study. The Centre for Maternal, Fetal, Newborn and Child Health Care Strategies, University of Pretoria helped to contact and distribute the email invitations to senior managers in Tshwane Health District. Out of the 26 managers invited, 24 participated in the ranking exercise resulting in a response rate of 92.3%. Information on HTA, the CM, and the objective of the assessment was prepared and presented to the managers in advance (2 May 2019). The objective of the workshop was to select and rank the AEs according to the relevance of the evidence needed for informed decisions. One author (DM) and trained researchers from the Centre facilitated the meeting and the ranking exercise. For each domain and AE, a 5-point Likert scale was applied, where “1” was least relevant and “5” was most relevant. Median scores were calculated per choice to characterize the category above and below which 50 percent of the scores fall. Interquartile ranges (IQR) were calculated to evaluate the degree of consensus per choice. Ratings with a median of <  = 2 and a narrow IQR (between 1 and 2) were considered to have reached consensus on the least relevant. A median of >  = 4 and an IQR between 4 and 5 were considered to be most relevant (see Table 1).

**Table 1:** Ranking of HTA Domains and assessment elements

Element ID	Topic/Issue	Median (IQR)	No of responses (%)
Health problem and current use of the technology (CUR)		5 (4,5)	24
	Target population		
A0007	What is the target population in this assessment?	5 (4,5)	24
A0023	How many people belong to this target population	5 (3.75, 5)	24
	Target condition		
A0002	What is the health condition	5 (4,5)	24
A0003	What are the known risk factors?	5 (4,5)	24
A0004	What is the natural course of the health condition?	4 (4,5)	23
A0005	What are the symptoms or health condition for the patient?	4 (4,5)	24
A0006	What are the consequences of the health condition for the society?	4.5 (4,5)	22
A0009	What aspects of the consequences are targeted by the Umbiflow?	4 (4,5)	23
	*Current management of the condition*		
A0018	What are the other typical or common alternatives to the Umbiflow?	4 (3.5,5)	23
A0024	How is the health condition currently diagnosed according to published guidelines and in practice?	4 (4,5)	24
A0025	How is the health condition currently managed according to published guidelines and in practice?	4 (4,5)	24
	Utilization		
A0001	For which health conditions and populations, and for what purposes is the technology used?	4 (3,5)	24
A0011	How much are the technologies utilized?	4 (3,5)	24
F0001	Is Umbiflow a new, innovative mode of care, an add-on to, or modification of a standard mode of care, or a replacement of a standard mode of care?	4 (3,5)	24
Description and technical characteristics of technology (TEC)		4 (3,5)	24
Safety (SAF)		5 (4,5)	24
	Patient safety		
C0006	What are the consequences of false positive, false negative and incidental findings generated by using the technology from the viewpoint of patient safety?	4 (3,5)	24
Clinical effectiveness (EFF)		4 (4,5)	23 (95.8)
	Mortality		
D0001	What is the expected beneficial effect of intervention on mortality?	4 (4,5)	24
	Morbidity		
D0005	How does using Umbiflow affect findings of the health condition?	4 (4,5)	24
D1004	What are the requirements for accuracy in the context where the technology will be used?	4 (3,5)	24
	Change-in-management		
D0020	Does use of the test lead to improved detection of the condition?	4 (4,5)	24
D0010	How does the technology modify the need for hospitalisation?	4 (3,5)	24
	Benefit-harm balance		
D0029	What are the overall benefits and harms of the technology in health outcomes?	4 (3,5)	24
Costs and Economic aspect		4 (3.4,5)	24
	*Process-related costs*		
G0006	What are the costs of processes related to acquisition and setting up new technology?	4 (3,5)	23
G0007	What are the likely budget impacts of implementing the technology?	4 (3,5)	23
Ethical aspect		4 (4,5)	24
	Autonomy		
F0006	Is there a need for any specific interventions or supportive actions concerning information in order to respect patient autonomy when the technology is used?	4 (3.5,5)	23
F0007	Does the implementation or withdrawal of the technology challenge or change professional values, ethics or traditional roles?	4 (3,4.5)	23
	Legislation		
F0014	Does the implementation or use of the technology affect the realisation of basic human rights?	4 (3,5)	23
Organizational aspect		4 (3,5)	24
	Health delivery process		
G0001	How does the technology affect the current work processes?	4 (3,5)	24
G0100	What kind of patient/participant flow is associated with the new technology?	4 (3,5)	24
G0002	What kind of involvement has to be mobilised for patients/participants and important others and/or caregivers?	4 (3,5)	21
G0003	What kind of process ensures proper education and training of staff?	4 (3,5)	23
	Management		
G0008	What management problems and opportunities are attached to the technology?	4 (3,5)	24
Patient and social aspect		4 (4,5)	23 (95.8)
	Patient’s perspectives		
H0100	What expectations and wishes do patients have with regard to the technology and what do they expect to gain from the technology?	4 (3,5)	24
	Communication aspects		
H0202	How are treatment choices explained to patients?	4 (3,5)	24
Legal aspect		4 (3.5,5)	23 (95.8)
	Autonomy of the patient		
I0034	Who is allowed to give consent for minors and incompetent persons?	4 (3,5)	24
	Privacy of the patient		
I0009	What do laws/binding rules require with regard to appropriate measures for securing patient data and how should this be addressed when implementing the technology?	4 (3.75,4.25)	24

### Step 2: Determining the decision criteria of hospital CEOs

The CEOs of the hospitals enrolled in cohort studies on Umbiflow™ were invited to identify the decision criteria they considered necessary for technology adoption. While the CM guides data collection and analysis, it does not extend to ways of translating the assessment results into decisions. In this study, it was envisioned that identifying a set of decision criteria early in the assessment process could ensure that the information needs of the hospital decision-makers are met using best available evidence. The fifteen components of the EVIDEM (Evidence and Value: Impact on Decision Making) framework for healthcare decision-making [23] were chosen as the basis. Broadly, these components, which are based on the intrinsic and extrinsic value of healthcare intervention includes need, improvement of outcomes and feasibility of application. To ensure conceptual consistency in the study, the criteria were first juxtaposed with the CM and found not to differ substantially in terms of structure, logic, and content. Currently, cohort trials are ongoing at nine sites in the eight provinces in South Africa. CEOs at the district hospitals in these provinces were asked to agree or disagree with each criterion in the framework (see “Additional File 1: Table S1a”).

The principal investigator of the cohort studies (RP) facilitated the distribution of the email invitation. The email included a cover letter inviting the CEOs to participate and described the study objectives and the list of criteria. The invitation stated that the information obtained will be used to prepare guidance towards developing an assessment tool and will be published. Invitations were sent by mail to all nine-study sites; the email communication and collection of responses took place between June and September 2019. Three out of the nine CEOs invited agreed to participate and returned filled-out questionnaires to the authors.

### Step 3: Identification of published studies on Umbiflow™ and continuous-wave Doppler ultrasound

While steps 1 and 2 served to define the scope and main principles of the assessment process, steps 3 and 4 aimed to collect evidence targeting Umbiflow™. In Step 3, the following databases were searched using search strategies based on the PICOTS framework delineated below: PubMed (MEDLINE), EMBASE, HTA CRD, and the INAHTA database.

- Population: Target population in these regions – low-risk singleton pregnant patients of > 28 weeks gestation or with symphysis fundal height (SFH) measurement of 26–30 cm if gestational age was unknown.
- Intervention: Use of continuous-wave DUS.
- Comparator: No screening using ultrasound
- Outcome: patient-relevant outcomes (e.g. early detection of anomalies; Increase in diagnostic accuracy compared to SFH measurement)
- Time: open to September 2019
- Study types: No restrictions to the type of study

First, published literature on continuous-wave DUS and its assessment was searched using the key words linked with Boolean operators. The search results were screened by one author (DM) and information was extracted from included studies based on a semi-structured information collection guide. After expert consultation (Step 4), this step 3 was revisited as additional criteria such as “low-risk” AND “third trimester” were included. Further targeted searches were conducted to identify potentially missed literature specifically on the technology’s technical properties, safety aspects, and application. The reference lists of all identified sources were searched for further relevant evidence.

### STEP 4 Collating user experience and expert consultation

Two authors who are also clinical investigators on Umbiflow (RP and TMH), provided unpublished data, additional literature, and direct expertise to complement the evidence generated by the literature search. The intent was to collate information on technical features, the health problem, and clinical practice using a structured tool (see “Additional File 1: Table S1c”).

Furthermore, nurses and clinicians participating in the cohort studies of Umbiflow™ were requested to share their experience. The questionnaire (see “Additional File 1: Table S1d”) to the nurses consisted of a section inquiring about their experience of using the DUS and on potential patient benefits and harms. The questionnaire to the clinicians (see “Additional File 1: Table S1d”) varied slightly to reflect their expertise and included additional sections on post-introduction management of the technology in comparison to standard care and the potential impact on the system. Both the questionnaires have been adapted from the NICE Medical Technologies Evaluation Program. A first version of the questionnaires was piloted with two clinicians to review coherence and appropriateness of content, language, and format. The refined version was then sent by mail to all study sites between June and September 2019. The research centre facilitated the distribution. During a site visit to one of the health care facilities using Umbiflow™, one of the authors (DM) also gathered information on patient satisfaction indirectly, with the nurse or clinician on duty asking patients about their satisfaction with the screening.

## Results

### Selection of domains and assessment elements under consideration

Twenty-four district health managers from the Tshwane Health District participated in scoring the domains and AEs. One to three managers did not provide scores for some of the criteria. The median, IQR, and the number of responses for each domain and sub-category AEs are presented in Table 1.

The domains on “health problem and current of use of technology” (CUR) and “safety” (SAF) had a median of 5 and the rest of the 9 domains had a median of 4. However, there was a variance observed in the IQR. 5 of the 9 domains including CUR and SAF reached a consensus with a strong agreement (IQR between 4 and 5) on their relevance. In the case of the other domains, the IQR varied between 3 and 5. For instance, managers were uncertain about the necessity of evidence on organizational implications (ORG) and questioned the appropriateness of detailed technical evaluation (TEC).

Variation was also observed across AEs within the individual domains (see Table 1). For instance, the disagreement on the relevance of some of the AEs may stem from the priority given to access to high quality maternal and child health and the relatively early phase of implementation of the device (cohort studies in different provinces). Similarly, even though the economic impact and affordability were relevant among the decision-makers (median = 4), the managers disagreed widely on the relevancy of individual AEs. This could be due to the feasibility of conducting an economic evaluation based on the current evidence.

### Selection of criteria for decision on adoption of a technology in the primary healthcare setting

The three CEOs of district hospitals unanimously agreed that disease severity, improvement in efficacy and effectiveness, and public health issues are important criteria for decision-making. All agreed on the economic impact of the intervention, affordability, and opportunity costs. Additionally, contextual factors such as the scope of the healthcare system, appropriate use of the intervention, and system capacity received a positive response from all.

The size of the affected population, or improvement in safety or tolerability, or historical and political context, or pressures from stakeholders or individuals, in the context surrounding healthcare intervention received two positive responses each. Respondents disagreed (2 to 1) on the importance of improvement in-patient reported outcomes and contextual criteria, such as priority of the population and equity of access as relevant decision criteria. The responses are shown in Table 2.

**Table 2:** Decision Criteria preferred by the hospital managers

Domains/criteria	Domains/criteria	(Frequency) Y/N
Need for the intervention	Disease severity	(3)/(0)
	Size of affected population	(3)/(0)
Outcomes of the intervention	Improvement of the effectiveness/efficacy	(2)/(0)
	Improvement safety/tolerability	(3)/(0)
	Improvement patient-perceived health/patient-reported outcomes	(2)/(1)
Type of benefit of the intervention	Interest towards public health	(3)/(0)
	Type of clinical benefit	(3)/(0)
Economic impact of intervention	Impact on the budget	(3)/(0)
	Impact on other spending—other medical/non-medical costs	(3)/(0)
Contextual criteria	Mandate and scope of healthcare system	(3)/(0)
	Population priority and access	(2)/(1)
	Common goal and specific interests	(1)/(1)
Feasibility of contextual criteria	System capacity and appropriate use of intervention	(3)/(0)
	Political / historical / cultural context	(1)/(1)
Opportunity Cost	Opportunity costs and affordability	(3)/(0)

### Collection of evidence from the four steps

The evidence map (Table 3) illustrates the evidence collected from steps 3 and 4 in the methodology.

**Table 3:**
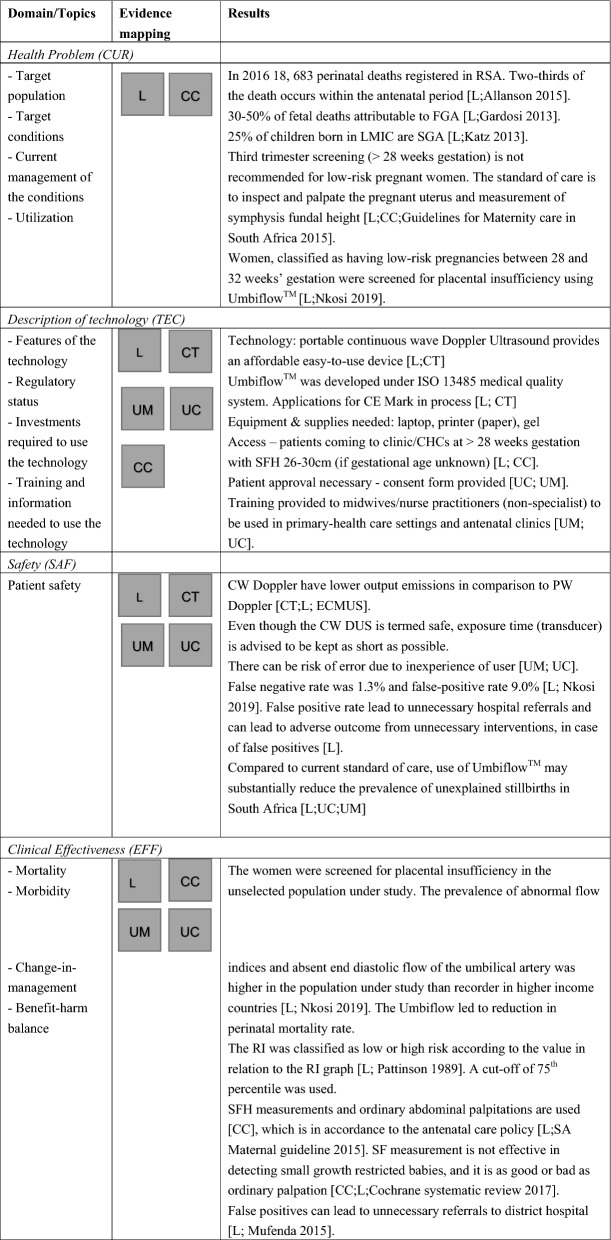

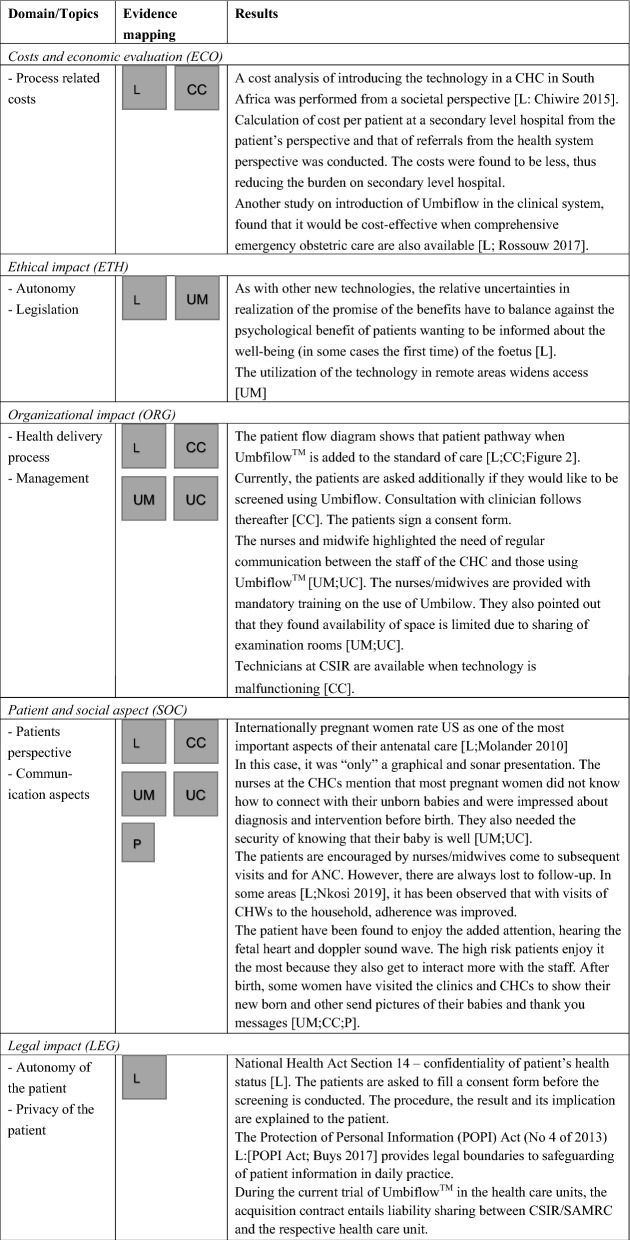
Decision- and policy-makers requirement to support decision

*L* Literature on health care system, legislation in the country, continuous wave Doppler ultrasound and Umbiflow™_;_ *CC* Clinician Consultation, *CT* technical consultation, *UM* user survey midwives and nurses, *UC* user survey clinician, *P* patient, *CW* continuous wave, *PW* pulse wave, *DUS* Doppler ultrasound, *U* umbiflow, *CHC* Community Health Centres, *ANC* antenatal care, *CHW* community health worker, *SAMRC* South African Medical Research Council, *CSIR* Council of Scientific and Industrial Research

In Step 3, a total of 65 relevant sources of evidence were collected following the search in bibliographic databases, expert consultation and hand searching the reference lists of already identified sources. The flow of information is documented in a PRISMA flowchart (see “Additional File 1: Fig. 1f”).

For all domains, a combination of evidence from peer-reviewed and grey literature with expert input was necessary to complete the evaluation of Umbiflow™. Evidence on the health problem and current use of the technology (CUR) was mainly obtained from global and local literature on maternal and child health (Step 3), supplemented by information provided during expert consultation (Step 4). Information on the technical characteristics (TEC) of the continuous-wave DUS in general and Umbiflow™ in particular was retrieved from technical literature and in consultation with the experts; users (Step 4) provided information on training needs and characteristics. For safety (SAF), the expert consultation yielded additional details on usability and type of errors. Evidence on economics (ECO) was obtained from the completed Master’s thesis on cost analysis and a conference abstract (paper unpublished) on cost-effectiveness analysis, shared by the PI. The site visit described in the methodology (Step 4) gave further insight into communication requirement and patient acceptance of the technology. Information on legal matters (LEG) was difficult to obtain; the two acts mentioned in Table 3 provided important background information on safeguarding patient rights.

### Users’ experience on Umbiflow™

The detailed responses provided by the nurse practitioners, midwives and clinicians through the survey questionnaire provided insight on the use of the Umbiflow™ for obstetric services in its intended setting in the South African health system. Eight nurses and six clinicians responded to the survey.

The nurses informed that utilization of these additional Doppler ultrasound services led to complications in work processes, misunderstandings and difficulties arising from having to share the examination rooms limiting privacy for patients. They recommended that trainees start using the device right after the training period to minimize reading errors. Computer literacy is mandatory, as sometimes assistance in dealing with computer malfunctioning can take time. They also pointed out the communication flow among doctors and nurses on duty at the centres should be maintained, as currently the technology is not included in the standard of care.

Clinicians welcomed an innovative solution, effective and low cost, addressing the need to prevent stillbirths. However, not all clinicians used it themselves. One of the clinicians perceived the use of Umbiflow™ as beneficial for patient care and early management. The technology had the potential to improve the current patient pathway by earlier recognition of fetus at risk for growth restriction at the local clinic level. Another clinician indicated that this could help in reduction of serious adverse events and resulting litigations. They were uncertain about the real cost of the technology when adopted into standard care and disagreed on the need for extra staff. A few suggested that the midwives currently employed could be trained on using the technology. A clinician stated that computer literacy is mandatory and another indicated that funding could be an influencing factor. Overall, they found that it would be beneficial at the PHC setting.

A mapping of the evidence according to the CM assessment elements is shown in Table 3.

### Evaluation of Umbiflow™

The following sections summarize the best available evidence on Umbiflow™ for the primary care setting in South Africa. Diagnosis using continuous wave DUS was evaluated and compared with usual care i.e. non-use of DUS or other forms of US**.**

### Health problem and current use of the technology (CUR)

In South Africa, unexplained fetal mortality is the largest single contributor to perinatal deaths [24]. A quarter of these fetuses are small for gestational age (SGA), two-thirds of these deaths occurs during the last trimester of pregnancy [25]. At the public PHC level, Community Health Centres (CHCs) often include a clinic for the local catchment area, a referral section with specialists, and a 24 h unit with maternity and casualty staff. Clinics draining into the Community Health Centres (CHCs) provide antenatal care for low and intermediate-risk women, including point of care blood and urine testing [26]. CHCs provide 24-h comprehensive health service with an obstetric unit run by midwives.

Currently, Umbiflow™ is used in the context of the ongoing cohort trials. After receiving their routine ANC visit, the women would be taken by the nurse or midwife to an examination room for the scan to be performed. The procedure of the examination is explained to the patient by either the nurse or the clinician present and the patient is asked to sign a consent form. Due to the high burden and abundant research, evidence on the health problem was readily available from the literature on maternal and child health care, as also highlighted in other studies [27]. However, information on the use of the technology was only forthcoming from experts involved in related trials.

### Description and technical characteristics (TEC)

Umbiflow™, is a continuous-wave DUS device for use at PHC facilities and antenatal clinics. The software processes the US signals to generate a high quality waveform depiction of the umbilical blood flow. The waveforms are displayed on the computer screen. The “resistance index” (RI) which can be directly linked to the functioning of the placenta [28] is calculated and plotted on a percentile chart against gestational age [29]. Regular training is provided to the staff using the technology. Evidence collated from the literature and in consultation with the clinicians and nurses underlined, the need of additional investments and mandatory training required to use the technology. Important services such as availability of maintenance contract, upgrade of software, regular technical analysis, and risk assessment are provided by CSIR. However, as the nurses indicated challenges such as power outage at the clinic, malfunctioning of the Umbiflow™ or delay in live technical support, exists in practice.

### Safety (SAF)

DUS can produce biological effects such as tissue heating representing a potential health risk. However, the continuous-wave DUS have low output intensity in comparison to pulse wave Doppler [30]. Nonetheless, it is advised to keep exposure time as short as possible [10].

The potential disadvantages include false-positive and false-negative results, although these rates are typically low [28, 31]. If a false positive occurs, the patients are likely exposed to avoidable cost and anxiety, which may lead to inappropriate intervention [32]. The false negative cases may not be captured unless the clinical condition of the mother changed [31].

### Evidence on clinical effectiveness (EFF)

Evidence on effectiveness of continuous-wave DUS was mainly obtained from literature, supplemented by information specifically on Umbiflow™ obtained from consultation with experts and users. All studies on Umbiflow™ were cohort studies, conducted in South Africa; randomized trials have not been conducted.

The studies on Umbfilow™ reported diagnosis related adverse events. Nkosi et al. [28] determined the false-negative rate in a sample of 226 low-risk pregnant women, by using Umbiflow™ and re-tested with conventional ultrasound and pulsed Doppler. A false negative rate of 1.3% and specificity of 98.7% in this sub-set was obtained for Umbiflow™. The study had a sensitivity of 91%. The three women in the study attended the high-risk clinic for full assessment [28]. In this study, the false positive rate was 9.0% with a sensitivity of 91.0 for 32/355 high risk cases. These women were referred back to their local clinic to continue with their routine ANC.

In 2005, Theron et al. [29] examined the effectiveness of the PC-based Umbiflow™ in relation to a well-known commercial standard system in a cohort study and found their accuracy to be comparable. Hugo et al. [31] evaluated the use of Umbiflow™ by trained midwives at a secondary hospital to assess the umbilical artery velocity waveforms in high-risk pregnancy. It was initially aimed at reducing unnecessary referrals for pregnant women with fetuses, which were SGA [33]. Findings showed that this continuous-wave DUS was effective in screening low-risk pregnancies, identifying at-risk fetuses requiring interventions, and in reduction of additional tests arising from false negative results [28].

Timely detection of abnormalities in the blood flow in the uterine artery followed by management of undiagnosed placental insufficiency could lead to prevention of perinatal deaths [6, 28, 34]. Appropriate management will depend on the severity and the gestation of the pregnancy. When it first presents at later stages of pregnancy (at 34 weeks), a scheduled C-section is a necessary action. As this can occur preterm, added risk of mortality and morbidity arises. Currently, no effective antenatal therapy exists for fetal growth restriction; hence, delivery could be the only viable option [35]. A recent study observed a 1.5% prevalence of absent end diastolic flow (AEDF), a sign of fetal vascular stress, in a population of low-risk pregnancies, indicating that using the Doppler RI information in the study population reduced the rate of macerated stillbirth by 60% [28, 34].

### Economic impact—costs and resources used (ECO)

The evidence for economic impact of use of DUS in a clinical setting was taken (see Section on evidence retrieval, above) from grey literature. Rossouw et al. [36] showed that introducing Umbiflow™ in South Africa would be cost-effective only with concurrently available comprehensive emergency obstetric care. They found the use for testing placental insufficiency to be more cost-effective than other interventions such as therapeutic feeding or antiretroviral treatment or new vaccine in South Africa [34]. Chiwire [32] performed a cost analysis of introducing Umbiflow™ in a community health centre in South Africa from a societal perspective and included consumables, personnel costs, and costs such as opportunity costs arising from visiting healthcare facilities. The study undertook calculation of cost per patient at a secondary level hospital from the patient’s perspective and referrals from the health system perspective. The societal cost at the PHC setting was approximately 3 times less than that at the secondary hospital, reducing the burden on secondary level health system.

### Organizational aspects (ORG)

The literature on Umbiflow™ and consultation with the users and experts provided evidence on organizational aspects. Healthcare professionals including nurses or, midwives can use the device after receiving short training to detect correct soundwave and visually interpret a correct wave pattern on the computer screen [33]. The learning curve is dependent on image interpretation and acquisition skills. Since the midwives or nurse practitioners receive a few days of mandatory training initially, the curve shows an early rise, followed by a plateau.

**Fig. 2:**
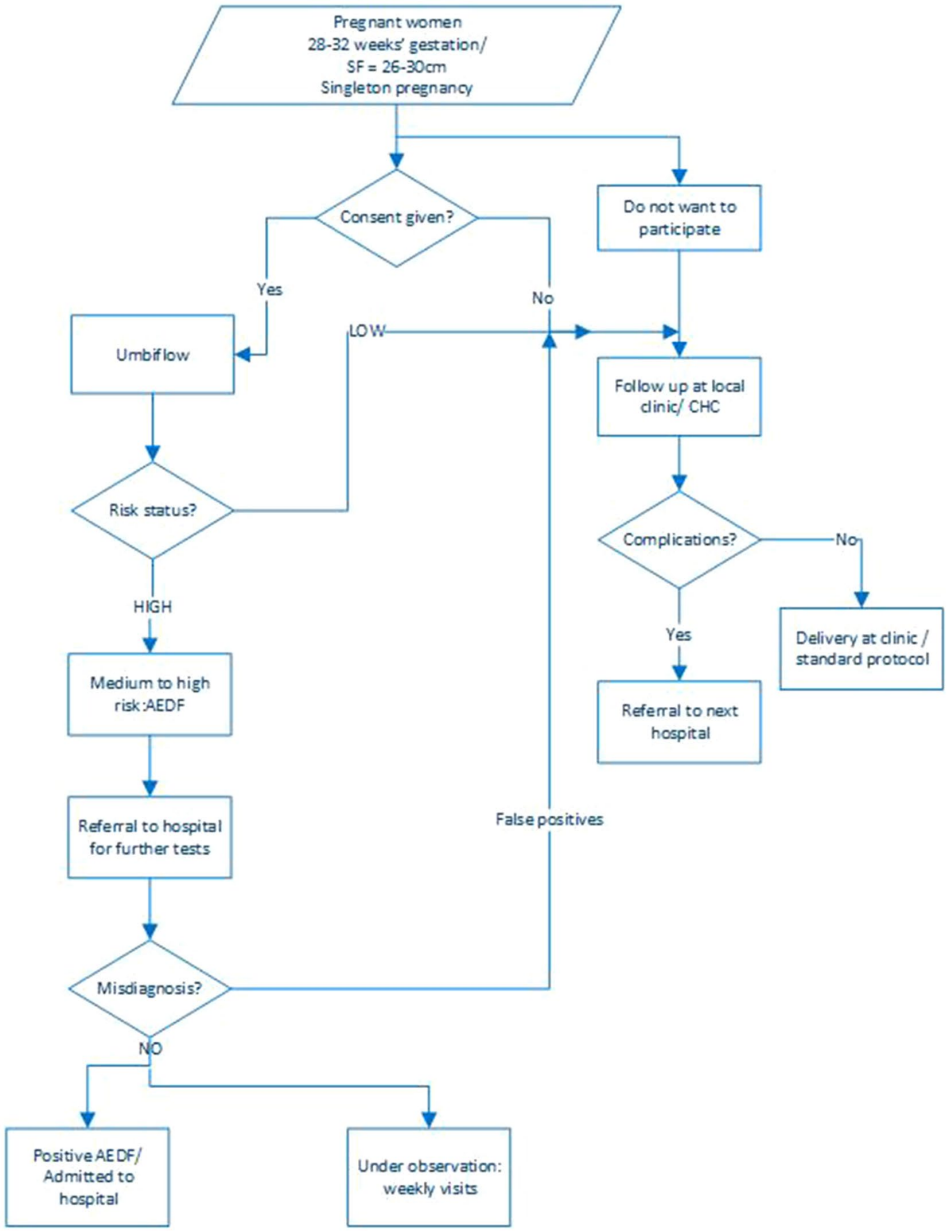
Patient pathway on introduction of Umbiflow™ into the clinical system in South Africa. Adopted from Nkosi et al. [28]

Information provided from current users of the technology in South Africa suggests that they sometimes lack resources to provide services in informative and proper manner (blood pressure monitor, furniture, scale, poor network connection, power shortage etc.). Additional infrastructure, such as extra examination room, is not required due to the portability feature of the machine, and screening can be integrated within the organizational infrastructure associated with standard care (see Fig. 2). However, this may not be always ideal due to a lack of privacy and insufficient hygiene conditions. Active interaction and communication between the routine clinic staff and health professionals using Umbiflow™ would ease organizational challenges. Nurses interviewed for this study suggested sensitization on the benefit of using Umbiflow™ among health professionals and the patient and caregiver through workshops or information days.

### Legal aspects (LEG)

Section 14 of the National Health Act [14, 37] of South Africa states the information relating to the patient’s health status is confidential. It further specifies that patients have to actively consent to any disclosure of their information. In the study setting, patients fill a consent form; the Umbiflow™ procedure, the result and its implications are explained to them. The Protection of Personal Information Act (No 4 of 2013) [38] provides legal boundaries to safeguard patient information in daily practice. No further legal implications of the introduction of Umbiflow™ could be identified.

### Ethical aspects (ETH)

The evidence on ethical aspect of using Umbiflow™ was extracted from literature on DUS. The use of Umbiflow™ is expected to lead to detection of anomalies in the third trimester. As with other ultrasounds, the relative uncertainties in realization of the promise of the benefits have to balance the psychological benefit of patients wanting to be informed about the well-being (in some cases the first time) of the fetus. This subjective benefit must be weighed against inconvenience of situations leading to unneeded referral to a district hospital or can lead to adverse outcome from unnecessary interventions, in case of false positives. The Umbiflow™ screening is done only with patient consent. Not all pregnant women visiting the clinics are willing to be screened, which could be due to religious, cultural, or spiritual beliefs [39–41].

### Patient and social aspects (SOC)

Patients are required to provide their consent before Umbiflow™ examination. Nurses at the CHCs confirmed that patients favoured the technology. They stated that, most patients were not knowledgeable on how to care for their unborn babies (e.g. intake of nutrients) and connect with them. The patients were impressed about the possibility of diagnosis and intervention before birth and some wished for an examination.

## Discussion

This study reports on the value of stakeholder engagement and consultation during scoping evidence. It tests the feasibility of using the EUnetHTA Core Model in a non-European country to assess continuous-wave DUS technologyfor adoption in low resourced PHC setting and highlight the potential organizational impact and clinical benefit of a simple low-cost technology in such a setting.

First, consensus was sought on the relevancy of the HTA CM domains and the guided questions. Briefly, the domains, CUR and SAF and three others, EFF, ETH and SOC had received a median of 5 and 4 respectively and a narrow IQR (4,5). The result of the ranking led to four other domains having a wider IQR (= > 3.4,5). Since some of the issues within these domains had a narrower IQR, these issues were considered. Since the model has 134 assessment elements, the final number selected was smaller, and the scope was more targeted. As with other adaptations, few of the issues were rearranged as they were viewed suitable in a different domain [42].

The scoring could have been influenced by the fact that the health services are free of charge for maternal, child healthcare and procurement and financial administration of PHC falls under district health office. Consensus was reached with a wider IQR (e.g. 3 to 5) for the evidence needed for organizational or legal aspects, as well as technical characteristics. This may be because the expression “technical characteristics,” is misleading for decision-makers. Due to the newness of the HTA concept and the CM among these stakeholders, misunderstanding and misjudgment of the selection could have resulted in error in the ranking of the domains and the respective AEs.

Second, this work sought to understand the decision process for procurement of the technology (i.e. hospital CEO). Generalization of the decision criteria is not possible at this stage as only three hospital managers responded to the questionnaire; however, their informational needs seem to be largely covered by the domains prioritized in this study. The AdHopHTA (Adopting hospital-based HTA) project, which is a European Project on hospital-based HTA aims to promote collaboration of hospital-based HTA initiatives. Unlike the recommendation given in the AdHopHTA guideline [43] consensus on the impact of political and strategic aspect of adoption of the technology was not reached. It is noteworthy that while the district health managers were uncertain about the relevance of organizational aspects, such as system capacity and appropriateness, both hospital CEOs and Umbiflow™ operators highlighted their significance. This further supports the main hypothesis of this study, namely that involving a broad range of stakeholders is crucial for understanding the scope of necessary evidence for informed decision-making.

Third, the inclusion of experts and users of the technology in a standardized manner in the evidence scoping process reduced the gaps in identified evidence, adding to the understanding of the appropriateness, acceptability, accessibility, and perceived affordability of the technology in its intended setting of application.

Fourth, the synthesis of collected evidence shows that screening using continuous-wave DUS may be better suited than only SFH measurements in detecting pregnancies at risk of stillbirth. Heazell et al. [44] in their study on economic and psychosocial consequences of stillbirth concluded that even though costs of stillbirth prevention is high, the combined direct, indirect and intangible costs are higher. WHO in its ANC guideline calls for low-cost and accurate screening tool for detecting abnormal fetal growth in LMICs [6]. Yet, a recent study of two-stage routine conventional ultrasound screening in LMICs detected no effect of screening on stillbirth prevention [7, 45]. This seriously questions the role of conventional ultrasound screening for prevention of stillbirths and calls for a randomized trial using Umbiflow™ to confirm its role in detection of at-risk pregnancies. The Department of Health of South Africa is supporting a project to implement Umbiflow™ as a routine screening tool for ANC.

## Strengths and limitations

The assessment carried out in this study demonstrates the promise of Umbiflow™ in context by drawing on additional, qualitative judgments on the functioning of the device, its proposed benefits, and the plausibility that its adoption will provide the claimed improvements. To mitigate uncertainty in the absence of robust randomized control trials, experts were consulted, and stakeholders involved, which aided the assessment process. However, it is important to reiterate the limitations of expert opinion as evidence for HTA, given its substantial potential for bias.

Involving the decision-makers on the ranking of the domains and the criteria for assessment sensitized them on the usefulness of CM and their acceptance on the relevancy of the evidence. The necessity of broader upstream consideration, besides, cost-effectiveness early on in the technology lifecycle [46] can contribute to the sustainability of the health system.

One of the advantage of Umbiflow™ is that an experienced sonographer is not needed. However, skill levels and scanning method may differ between the operators and may need the intervention of the clinician on duty. However, use is simple for the nurses and they could master the techniques within short time.

The selection of assessment domains and elements based on deliberation is challenging due to the diversity of user demands and preferences. Furthermore, none of the district managers and hospital decision makers involved in this study requested further clarifications or information. It is therefore uncertain how these stakeholders interpreted the description of the individual AEs. Due to the complexity and newness of the CM, some respondents may have misunderstood and not selected certain relevant AEs.

Since 2005, further development and improvement of the technology incorporating latest mobile and information technologies, software development has taken place. However, no studies have compared previous models to the current models and therefore it is unclear whether results obtained from previous models are generalizable to the model currently under study.

This study did not consult gynecologists who are unfamiliar with continuous-wave DUS or do not work in the PHC setting. They could have provided their perspective on the benefit of a continuous-wave DUS to be used at the PHC level.

Involvement of the research institute funding the project may have the potential for introducing bias in the reporting of outcomes.

## Conclusion

This study tested the applicability of the HTA CM to evaluate the value of a continuous-wave DUS, the Umbiflow before adoption in a low-resource setting. Mapping a set of decision criteria before the assessment process addressed the perceived gaps in evidence. Collection and generation of evidence based on the HTA CM and the chosen decision criteria provided a generalized but structured guidance on the methodology. Several issues did not apply for the technology and the context of its use. To streamline the issues and thus the assessment elements, careful appraisal and application to other classes of MDs to eliminate those that do not fit into the African context will be required.

Engaging and consulting stakeholders locally was imperative to understand the context, reduce evidence gaps and address the uncertainties in the evidence, ultimately paving the way for technology adoption. Transparency in the assessment process and method is vital, as the assessment involved experience and elements of judgement. The consideration of risks associated with continuous-wave DUS use and its low cost need to be balanced with the acceptance and the promise of the technology in the context. Post-introduction, further robust evidence and its analysis will be essential to determine the realised value and thus the future use of this technology, which is competing with similar innovative technology.

## Supplementary Information

**Additional file1: Table S1a**. Step 1: Identification of criteria necessary to decide on adoption of a technology in the hospitals. **Table S1b**. STEP 2: Identification of elements necessary to assess Umbiflow. **Table S1c**. STEP 4: Consultation of clinical and technical expert on Umbiflow. **Table S1d**: STEP 5: User Questionnaire. **Fig. S1f**: Prisma Flowchart (DOCX 84 KB)

## Data Availability

The data used in this evaluation are available from the corresponding author on reasonable request.

## Abbreviations

AEDF: Absent end diastolic flow
AE: Assessment element
ANC: Antenatal care
CEO: Chief executive officer
CHC: Community health centre
CM: Core Model
CSIR: Centre for Scientific and Industrial Research
CUR: Health problem and current of use of technology
DUS: Doppler ultrasound
ECO: Economic impact
EFF: Clinical effectiveness
ETH: Ethical aspects
EUnetHTA: European Network for Health Technology Assessment
EVIDEM: Evidence and Value: Impact on Decision making
HTA: Health Technology Assessment
IQR: Interquartile range
LEG: Legal aspects
LMICs: Low- and middle- income countries
MD: Medical device
ORG: Organizational aspects
PHC: Primary healthcare
PI: Principal investigator
RI: Resistance index
SAF: Safety
SAMRC: South African Medical Research Centre
SFH: Symphysis fundal height
SGA: Small for gestational age
SSA: Sub-Saharan Africa
SOC: Patient and social aspects
TEC: Technical characteristics
UHC: Universal health coverage
