# Supplementary material for: Portable continuous wave Doppler ultrasound for primary healthcare in South Africa: can the EUnetHTA Core Model guide evaluation before technology adoption?
Source: Cost Eff Resour Alloc. 2021 Feb 15;19:8. doi: 10.1186/s12962-021-00261-z (PMC7885360; doi:10.1186/s12962-021-00261-z)
Supplement: Supplementary file 1 — Additional file1: Table S1a. Step 1: Identification of criteria necessary to decide on adoption of a technology in the hospitals. Table S1b. STEP 2: Identification of elements necessary to assess Umbiflow. Table S1c. STEP 4: Consultation of clinical and technical expert on Umbiflow. Table S1d: STEP 5: User Questionnaire. Fig. S1f: Prisma Flowchart (DOCX 84 KB) [file 12962_2021_261_MOESM1_ESM.docx]

**Additional File 1**

**Supplementary Table 1a**. Step 1: Identification of criteria necessary to decide on adoption of a technology in the hospitals

**Supplementary Table 1b.** STEP 2: Identification of elements necessary to assess Umbiflow

**Supplementary Table 1c:** STEP 4: Consultation of clinical and technical expert on Umbiflow

**Supplementary Table 1d:** STEP 5: User Questionnaire

**Supplementary Figure 1f:** Prisma Flowchart

**Supplementary Table 1a. Step 1: Identification of criteria necessary to decide on adoption of a technology in the hospitals**

Adapted from EVIDEM

| **DOMAINS / Criteria** | ***Possible sub-criteria*** | **Definitions** | **Should be considered?** |
| --- | --- | --- | --- |
|  | | | |
| **NEED FOR INTERVENTION** | | | |
| **Disease severity** | - *Effect of disease on life-expectancy* - *Effect of disease on morbidity (includes disability and function)* - *Effect of disease on patients’ quality of life* - *Effect of disease on caregivers’ quality of life* | Severity of the health condition of patients treated with the proposed intervention (or severity of the health condition that is to be prevented) with respect to mortality, morbidity, disability, function, impact on quality of life, clinical course (i.e., acuteness, clinical stages). | Y/N |
| **Size of affected population** | - *Prevalence* - *Incidence* | Number of people affected by the condition (treated or prevented by the proposed intervention) among a specified population at a specified time; can be expressed as annual number of new cases (annual incidence) and/or proportion of the population affected at a certain point in time (prevalence). | Y/N |
| **OUTCOMES of INTERVENTION** | | | |
| **Improvement of effectiveness/ efficacy** | - *Magnitude of health gain* - *Percentage of the target population expected to realize the anticipated health gain* - *Onset and duration of health gain* - *Sub-criteria for the measure of efficacy specific to the therapeutic area* | Capacity of the intervention to prevent or to produce a desired (beneficial) change in signs, symptoms or course of the targeted condition above and beyond beneficial changes produced by alternative interventions. | Y/N |
| **Improvement safety / tolerability** | - *Adverse events* - *Serious adverse events* - *Fatal adverse events* - *Short-term safety* - *Long-term safety* - *Tolerability* | Capacity of the proposed intervention to produce a reduction in intervention-related harmful or undesired health effects compared to alternative interventions. | Y/N |
| **Improvement patient-perceived health / patient-reported outcomes** | - *Improvement in health-related quality of life* - *Impact on autonomy* - *Impact on dignity* - *Convenience / ease of use / mode & setting of administration* | Capacity of the intervention to produce beneficial changes in patient-perceived health and patient-reported outcomes (PROs) (e.g., quality of life) above and beyond beneficial changes produced by alternative interventions; also includes improvement in convenience to patients. | Y/N |
| **TYPE OF BENEFIT OF INTERVENTION** | | | |
| **Interest towards public health** |  | Nature of the preventive benefit or risk reduction provided by the proposed intervention at the population-level (e.g., eradication, prevention, reduction in disease transmission, reduction in the prevalence of risk factors). Public health perspective. | Y/N |
| **Type of clinical benefit** |  | Nature of the clinical benefit provided by the intervention at the patient level (e.g., symptom relief, prolonging life, cure). | Y/N |
| **ECONOMICAL IMPACT OF INTERVENTION** | | | |
| **Impact on the budget** | - *Net cost of intervention* - *Acquisition cost* - *Implementation/ maintenance cost* | Net cost of covering the intervention (excluding other spending). This represents the differential between expected expenditure for the intervention and potential cost savings that may result from replacement of other intervention(s) currently covered by the health plan. Limited to cost of intervention (e.g. acquisition cost, implementation and maintenance cost, out-of-payment). | Y/N |
| **Impact on other spending– other medical/ non-medical costs** | - *Impact on primary care expenditures* - *Impact on hospital care expenditures* - *Impact on long-term care expenditures* - *Impact on productivity* - *Financial impact on patients* - *Financial impact on caregivers* - *Costs to the wider social care system* | Impact of the proposed intervention on other medical costs (excluding intervention cost) such as hospitalization, specialist consultations, adverse events costs, long-term care, out-of-pocket payment etc.  Impact of the proposed intervention on non-medical costs (excluding intervention cost) such as disability costs, social services, lost productivity, caregiver time, etc. | Y/N |
| **CONTEXTUAL CRITERIA** | | | |
| **Mandate and scope of healthcare system** |  | Alignment of the intervention with the mandate/scope of the healthcare system. The goal of healthcare is to maintain normal functioning. Mission and scope of healthcare plans/systems derive from this principle. | Y/N |
| **Population priorities and access** | - *Current priorities of health system (e.g., disabled; low socioeconomic status; specific age groups)* - *Special populations (e.g., ethnicity)* - *Remote communities* - *Rare diseases* - *Specific therapeutic areas* | Alignment of the intervention with current priorities of health system/plan. Priorities for specific groups of patients are defined by societies/decisionmakers and reflect their moral values. Such considerations are aligned with the principle of justice, which considers treating like cases alike and different cases differently and often gives priority to those who are worst-off. | Y/N |
| **FEASIBILITY CONTEXTUAL CRITERIA** | | |  |
| **System capacity and appropriate use of intervention** | - *Organizational requirements (e.g., process, premises, equipment)* - *Skill requirements* - *Legislative requirements* - *Surveillance requirements* - *Risk of inappropriate use* - *Institutional limitations to uptake* - *Ability to reach the whole target region/population* | The capacity of a healthcare system to implement the intervention and to ensure its appropriate use depends on its infrastructure, organization, skills, legislation, barriers and risks of inappropriate use. Such considerations include mapping current systems and estimating whether the use of the intervention under scrutiny requires additional capacities. | Y/N |
| **Political / historical / cultural context** | - *Political priorities and context* - *Cultural acceptability* - *Precedence (congruence with previous and future decisions)* - *Impact on innovation & research* - *Impact on partnership & collaboration among healthcare stakeholders* | The political, historical or cultural context may influence the value of an intervention with respect to specific political situations and overall priorities (e.g., priority for innovation) as well as habits, traditions and precedence. | Y/N |
| **Opportunity cost** | | | |
| **Opportunity costs and affordability** | - *Opportunity costs for patient (forgone resources)* - *Opportunity costs for population (forgone resources)* - *Affordability* | Consideration of the medical resources that may be forgone (opportunity costs) if the intervention is implemented and whether the healthcare system can afford implementing the intervention. Both affordability and opportunity cost considerations require a financial/budgeting exercise. Opportunity costs and affordability can be considered at the system/institution level and at the patient level. | Y/N |

**Supplementary Table 1b. STEP 2: Identification of elements necessary to assess Umbiflow**

**Health problem and current use of the technology**

| Topic | ID | Issue |
| --- | --- | --- |
| Target population | A0007 | What is the target population in this assessment? |
|  | A0023 | How many people belong to this target population |
| Target condition | A0002 | What is the health condition falling under the scope of this assessment? |
|  | A0003 | What are the known risk factors for the health condition? |
|  | A0004 | What is the natural course of the health condition? |
|  | A0005 | What are the symptoms or health condition for the patient? |
|  | A0006 | What are the consequences of the health condition for the society? |
|  | A0009 | What aspects of the consequences are targeted by the Umbiflow? |
| Current management of the condition | A0018 | What are the other typical or common alternatives to the Umbiflow? |
|  | A0024 | How is the health condition currently diagnosed according to published guidelines and in practice? |
|  | A0025 | How is the health condition currently managed according to published guidelines and in practice? |
| Utilization | F0001 | Is Umbiflow a new, innovative mode of care, an add-on to, or modification of a standard mode of care, or a replacement of a standard mode of care? |

**Safety**

| Topic | ID | Issue |
| --- | --- | --- |
| Patient safety | C0002 | What are the harms related to the technology? Are they due to dosage or frequency of application? |
|  | C0006 | What are the consequences of false positive, false negative and incidental findings generated by using the technology from the viewpoint of patient safety? |
|  | C0007 | Are the technology and comparator(s) associated with user-dependent harms? |
|  | C0004 | Does the frequency or severity of harms change over time or in different settings? How? |
|  | C0005 | Who are the susceptible patients who will more likely be harmed through the use of the technology? |

**Description and the technical characteristics of the technology**

| Topic | ID | Issue |
| --- | --- | --- |
| Features of the technology | B0001 | What is this technology and the comparator(s)? |
|  | B0002 | What is the claimed benefit of the technology in relation to the comparator(s)? |
|  | B0003 | What is the phase of development and implementation of the technology and the comparator(s)? |
|  | B0004 | Who administers the technology and the comparator(s) and in what context and level of care are they provided? |
|  | B0018 | Are reference values clearly established? |
| Regulatory status | A0020 | For which indications has the technology received marketing authorisation or CE marking? |
| Investment and tools required to use the technology | B0009 | What equipment and supplies are needed to use the technology and the comparator(s)? |
|  | B0008 | What kind of special premises are needed to use the technology and the comparator(s)? |
|  |  |  |
| Training and information need to use the technology | B0012 | What kinds of requirements in terms of qualification and quality assurance processes are needed for the use or maintenance of Umbiflow? |
|  | B0013 | What kinds of skills and training characteristics and information are needed for the personnel using the technology? |

**Clinical effectiveness**

| Topic | ID | Issue |
| --- | --- | --- |
| Mortality | D0001 | What is the expected beneficial effect of intervention on mortality? |
| Morbidity | D0005 | How does using Umbiflow affect findings of the health condition? |
| Health related quality of life | D0012 | What is the effect of (use of) Umbiflow on generic-quality of life? |
|  | D0013 | What is the effect of (use of) Umbiflow on disease-specific quality of life? |
| Patient satisfaction | D0017 | Were patients satisfied with the technology? |

**Economic impact**

| Topic | ID | Issue |
| --- | --- | --- |
| Measurement and estimation of outcomes | E0005 | What is(are) the measured and/or estimated health-related outcome(s) of the assessed technology and its comparator(s) (outcome identification, measurement and valuation)? |
| Examination of costs and outcomes | E0006 | What are the estimated differences in costs and outcomes between the technology and its comparator(s)? |

**Ethical aspects**

| Topic | ID | Issue |
| --- | --- | --- |
| Benefit harm balance | F0010 | What are the known and estimated benefits and harms for patients when implementing or not implementing the technology? |
|  | F0011 | What are the benefits and harms of the technology for relatives, other patients, organisations, commercial entities, society, etc.? |
| Autonomy | F0006 | Is there a need for any specific interventions or supportive actions concerning information in order to respect patient autonomy when the technology is used? |
|  | F0007 | Does the implementation or withdrawal of the technology challenge or change professional values, ethics or traditional roles? |
| Legislation | F0014 | Does the implementation or use of the technology affect the realisation of basic human rights? |

**Organizational aspects**

| Topic | ID | Issue |
| --- | --- | --- |
| Health delivery process | G0001 | How does the technology affect the current work processes? |
|  | G0100 | What kind of patient/participant flow is associated with the new technology? |
|  | G0002 | What kind of involvement has to be mobilised for patients/participants and important others and/or caregivers? |
|  | G0003 | What kind of process ensures proper education and training of staff? |
|  | G0004 | What kinds of co-operation and communication of activities have to be mobilised? |
|  | G0012 | How is the quality assurance and monitoring system of the new technology organised? |
| Structure of the healthcare system | G0101 | What are the processes ensuring access to the new technology for patients/participants? |
| Management | G0009 | Who decides which people are eligible for the technology and on what basis? |

**Patient & social aspects**

| Topic | ID | Issue |
| --- | --- | --- |
| Patient’s perspectives | H0200 | What are the experiences of living with the condition? |
|  | H0100 | What expectations and wishes do patients have with regard to the technology and what do they expect to gain from the technology? |
|  | H0002 | What is the burden on care-givers? |
| Communication aspects | H0202 | How are treatment choices explained to patients? |
|  | H0203 | What specific issues may need to be communicated to patients to improve adherence? |

**Legal aspects**

| Topic | ID | Issue |
| --- | --- | --- |
| Autonomy of the patient | I0002 | What kind of legal requirements are there for providing appropriate information to the user or patient and how should this be addressed when implementing the technology? |
|  | I0034 | Who is allowed to give consent for minors and incompetent persons? |
| Privacy of the patient | I0007 | Is there a possibility that the use of the technology produces additional information that is not directly related to the current care of the patient and may violate their right to respect for privacy? |
|  | I0008 | What do laws/binding rules require with regard to informing relatives about the results? |
|  | I0009 | What do laws/binding rules require with regard to appropriate measures for securing patient data and how should this be addressed when implementing the technology? |
| Equality in healthcare | I0011 | How are treatment choices explained to patients? |
| Authorisation and safety | I0015 | What authorisations and register listings does the technology have? |
| Ownership and liability | I0019 | What should be known about the intellectual property rights and potential licensing fees? |

**Supplementary Table 1c. STEP 4: Consultation of clinical and technical expert on Umbiflow**

Adapted from EUnetHTA medical devices evidence submission template short version

| Domains of the EUnetHTA Core Model | Adapted version used in stakeholder consultations included example questions |
| --- | --- |
| Health problem and current use of the technology | 1. What is the health problem and current clinical practice? 2. What is the burden of disease? 3. Describe the pathway of care that incorporates Umbiflow. 4. Provide a justification for the proposed positioning of the technology and the definition of the target population |
| Technical characteristics of Umbiflow | 1. What safety issues need to be considered when using Umbiflow?  2. What is the regulatory status?  3. What is the context and level of care?  4. State any contraindications or groups for whom Umbiflow is not recommended |
| Clinical effectiveness | How effective is Umbiflow in detection of fetal abnormalities? |

**Supplementary Table 1d. STEP 5: USER QUESTIONNAIRE**

Adapted from NICE Medical Technologies Evaluation Programme

**SISTERS’ QUESTIONNAIRE**

**Your information**

| **Name:** |  |
| --- | --- |
| **Job title:** |  |
| **Organisation:** |  |

**Please answer the following questions as fully as possible to provide further information about Umbiflow.**

| 1 | Please describe your level of experience with Umbiflow:   - Are you familiar with it? - Are you currently using it? - How many days/months have you been using it? |  |
| --- | --- | --- |

**Potential patient* benefits**

| 2 | What do you consider the potential benefits of Umbiflow to pregnant women? |  |
| --- | --- | --- |
| 3 | Does Umbiflow have the potential to change the current services to the pregnant population? |  |
| 4 | Does Umbiflow have the potential to change clinical outcomes? |  |
| 5 | Are any changes to facilities or infrastructure (example private rooms, extra furniture, computer etc) needed in order to use Umbiflow? |  |
| 6 | Does the use of Umbiflow require specific training? |  |
| 7 | Are you aware of any safety concerns surrounding Umbiflow? |  |

**General advice**

| 8 | Please add any further comments on your particular experience with or knowledge of Umbiflow or experiences within your organisation. |  |
| --- | --- | --- |

**Other considerations**

| 9 | What difficulties did you have with the usability or practical aspects of Umbiflow? |  |
| --- | --- | --- |
| 10 | How does Umbiflow affect your current work process at the clinic? |  |

**CLINICIAN QUESTIONNAIRE**

**Your information**

| **Name:** |  |
| --- | --- |
| **Job title:** |  |
| **Organisation:** |  |

**Please answer the following questions as fully as possible to provide further information about Umbiflow.**

| 1 | Please describe your level of experience with Umbiflow:   - Are you familiar with it? - Have you used it? Are there any issues? - Have you been involved in any research or development on Umbiflow? |  |
| --- | --- | --- |

**Current management**

| 2 | How innovative is Umbiflow, compared to the current standard of care? Is it a minor variation or a novel concept/design? |  |
| --- | --- | --- |
| 3 | Are you aware of any other competing or alternative technologies available to the health care system, which have a similar function/mode of action to Umbiflow?  If so, how do these products differ from Umbiflow? |  |

**Potential patient benefits**

| 4 | What do you consider the potential benefits of Umbiflow to pregnant women? |  |
| --- | --- | --- |
| 5 | Does Umbiflow have the potential to change (improve) the current pathway or clinical outcomes? |  |

**Potential system impact**

| 6 | What do you consider the potential benefits of Umbiflow to the health care and social care system? |  |
| --- | --- | --- |
| 7 | Considering the care pathway as a whole, including initial capital and possible future costs avoided, is Umbiflow likely to cost more or less than current standard of care, or about the same? |  |
| 8 | What do you consider the resource impact from adopting Umbiflow?  Could it, for example, change the number or type of staff needed, the need for other equipment, or effect a shift in the care setting such as from outpatient to inpatient, or primary to secondary care?  Are any changes to facilities or infrastructure, needed in order to use Umbiflow?  Are any specific training needed? |  |
| 9 | Is any specific training needed? |  |
| 10 | Are you aware of any safety concerns or regulatory issues surrounding Umbiflow? |  |

**General advice**

| 11 | Please add any further comments on your particular experience or knowledge of Umbiflow, or experiences within your organisation. |  |
| --- | --- | --- |

**Other considerations**

| 12 | Are you aware of any issues which would prevent Umbiflow from being adopted in your organisation? |  |
| --- | --- | --- |

**Supplementary** **Figure 1f: Prisma Flowchart**

Excluded (n=25)

Non Doppler
(n = )

Titles/abstracts screened (n=121)
(n = )

Records after duplicates removed
(n =121)

Additional records identified through other sources
(n=26)

## Identification

## Eligibility

## Included

## Screening

Studies on Doppler Ultrasound and Umbiflow
(n = 65)

Working of DUS, technical characteristics (n= 39)

Health problem, safety of US, social aspect, patient satisfaction (n =10)

Economic study (n=2)

HTA (n=2)

NICE Summary (n=1)

Umbiflow (n=8)

Cluster RCTs (n=1)

Cochrane review (n=2)

Full-text articles excluded
(n =31)

(high-risk pregnancy, first trimester only)

Full-text articles assessed for eligibility
(n = 96)

(English=95, German=1)

Electronic database searches: Medline, EMBASE, HTA CRD, and the INAHTA database (n = 253 records)

Records identified through database searching
(n =253)
